# Development of a Prodrug of Camptothecin for Enhanced Treatment of Glioblastoma Multiforme

**DOI:** 10.1021/acs.molpharmaceut.0c00968

**Published:** 2021-03-01

**Authors:** Elisa Checa-Chavarria, Eva Rivero-Buceta, Miguel Angel Sanchez Martos, Gema Martinez Navarrete, Cristina Soto-Sánchez, Pablo Botella, Eduardo Fernández

**Affiliations:** † Institute of Bioengineering, Universidad Miguel Hernández, Elche, Spain and Centre for Network Biomedical Research (CIBER-BBN), Avenida de la Universidad s/n, 03202 Elche, Spain; ‡ Instituto de Tecnología Química, Universitat Politècnica de València-Consejo Superior de Investigaciones Científicas, Avenida de los Naranjos s/n, 46022 Valencia, Spain

**Keywords:** glioblastoma multiforme, camptothecin, 5-aminolevulinic acid, blood−brain barrier, targeting

## Abstract

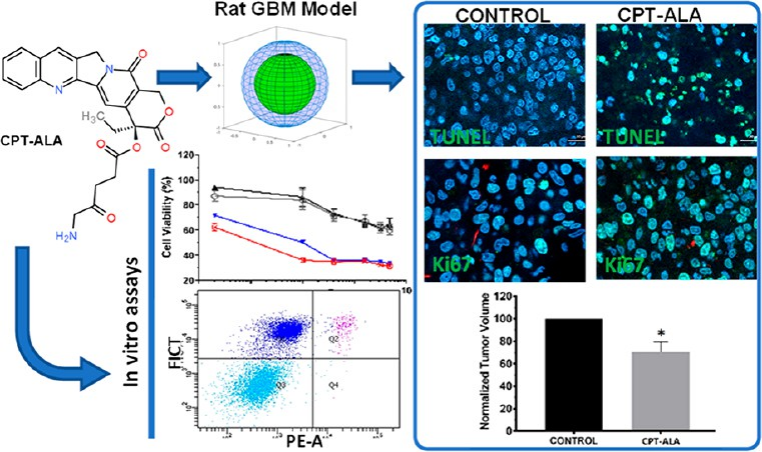

A novel therapeutic approach for glioblastoma multiforme (GBM) therapy has been carried out through *in vitro* and *in vivo* testing by using the prodrug camptothecin-20-*O*-(5-aminolevulinate) (CPT-ALA). The incorporation of ALA to CPT may promote uptake of the cytotoxic molecule by glioblastoma cells where the heme synthesis pathway is active, improving the therapeutic action and reducing the side effects over healthy tissue. The antitumor properties of CPT-ALA have been tested on different GBM cell lines (U87, U251, and C6) as well as in an orthotopic GBM model in rat, where potential toxicity in central nervous system cells was analyzed. *In vitro* results indicated no significant differences in the cytotoxic effect over the different GBM cell lines for CPT and CPT-ALA, albeit cell mortality induced by CPT over normal cell lines was significantly higher than CPT-ALA. Moreover, intracranial GBM in rat was significantly reduced (30% volume) with 2 weeks of CPT-ALA treatment with no significant side effects or alterations to the well-being of the animals tested. 5-ALA moiety enhances CPT diffusion into tumors due to solubility improvement and its metabolic-based targeting, increasing the CPT cytotoxic effect on malignant cells while reducing CPT diffusion to other proliferative healthy tissue. We demonstrate that CPT-ALA blocks proliferation of GBM cells, reducing the infiltrative capacity of GBM and promoting the success of surgical removal, which improves life expectancy by reducing tumor recurrence.

## 1 Introduction

Glioblastoma multiforme (GBM) is the most aggressive and lethal type of brain tumor, having a median survival of approximately 15 months. It is considered as level IV glioma by the World Health Organization.^1^ It is the most frequent type of primary tumor of the central nervous system (CNS), with 2–3 new diagnosis per 100 000 inhabitants every year.^1,2^ It has a characteristic genetic profile that favors a high level of mitotic activity and angiogenesis,^3^ inhibiting apoptosis and enabling a quick generation of resistance against treatment. It also preserves the capability to migrate from precursor cells, astrocytes, infiltrating into the cortical tissue and leading to metastasis in other regions of the CNS.^4,5^ Due to all these characteristics, GBM has the ability to grow and evolve faster than any other CNS tumor, increasing its malignant phenotype, poor prediction, and lethality. Moreover, the protection provided by the blood–brain barrier (BBB)^6^ converts GBM into one of the tumor types more difficult to cure and with stronger resistance.^7^

Current standard therapies include the surgical removal of as much tumor tissue as possible, followed by an adjuvant treatment with radiotherapy and chemotherapy, using temozolomide, also known as the Stupp protocol.^8^ Nevertheless, even with such aggressive treatment, the risk of recurrence is very high.^7^ Consequently, the average survival of patients is barely 15 months after diagnosis.^8,9^ When recurrence is present, the common treatment used is bevacizumab.^10^ The major advantage of temozolamide and bevacizumab is their capacity to move through the BBB, the major obstacle for the diffusion of chemotherapeutics agents into the CNS.^11^

In this context, camptothecin (CPT) is a natural pentacyclic alkaloid isolated from the oriental tree *Camptotheca accuminata* by Wall et al. in 1966,^12^ which is well-known for its antitumor activity against a wide spectrum of human cancers.^13^ CPT stabilizes via a noncovalent link the complex formed by the topoisomerase I and DNA during replication. As a result, the enzyme is inhibited and DNA structure is damaged, leading to apoptosis. Because of the mechanism of actuation of this drug, tumors with a high index of proliferation like GBM are more sensitive to CPT and its analogues.^14,15^ Unfortunately, CPT presents some major pharmacological limitations that preclude its clinical application, like poor water solubility and rapid lactone ring hydrolysis at physiological pH, leading to the inactive carboxylate form,^16^ and hampered diffusion through the BBB.

In an effort to improve CPT solubility and pharmacokinetics, different structural derivatives have been developed. Most derivatives of CPT have been obtained through the substitution of the quinolone ring. However, to date, only two of these CPT analogues have been approved for clinical use. These are irinotecan (CPT-11) and topotecan, which are able to cross the BBB,^17^ but their intrinsic activity is clearly lower than the pristine drug.^13^ Recent studies have been focused on different conjugates (ester, amide, carbonate, etc.) at the C20 position. These systems improve CPT delivery and bioavailability and may also introduce alternative administration routes for parenteral injection.^18−21^ Conversely, in order to use CPT against GBM, it is compulsory to improve the diffusion to the CNS.

In this scenario, our group recently described a CPT prodrug with 5-aminolevulinic acid (5-ALA): camptothecin-20-*O*-(5-aminolevulinate) (CPT-ALA).^22^ The rationale for using 5-ALA to synthesize the prodrug is based on the targeting and photodynamic properties of this molecule. First, 5-ALA is able to go through the BBB,^23^ which could favor the diffusion to the CNS of small molecules like CPT. Second, 5-ALA is a metabolic precursor of protoporphyrin IX (PpIX),^24,25^ a photoactive compound emitting red light at 635 nm when excited with 375–440 nm wavelength.^26^ In this context, it has been reported that 5-ALA uptake and PpIX synthesis are greatly increased in GBM,^27^ which enhances the targeting role of 5-ALA. Indeed, 5-ALA is used in clinical practice to locate the tumor in the brain, which enables precise surgical removal of GBM by fluorescence guided resection, maximizing tumor resection and having an important effect in improving patient healthcare.^28^ Finally, the endogenous PpIX accumulation promoted by 5-ALA increases tissue photosensitivity. For this reason, ALA has also been used in photodynamic therapy to treat several types of non-melanoma skin cancers,^29^ and its effect in GBM is also being investigated.^30^

In this work, we present a preclinical study of CPT-ALA prodrug as a novel therapeutic approach for GBM therapy, showing its anticancer and cytotoxic properties. The incorporation of 5-ALA to CPT follows a double strategy: first, promoting CPT diffusion through the BBB and then targeting the cytotoxic molecule to GBM cells, which should improve the therapeutic action, and in parallel, reducing the side effects over healthy tissue. For this reason we tested the antitumor properties of this prodrug on different GBM cell lines (U87, U251, and C6) as well as in an orthotopic glioblastoma model in rat while analyzing the potential toxicity over healthy tissue and CNS cells. Our results suggest that CPT-ALA prodrug shows high antitumor activity both *in vitro* and *in vivo*, without producing any neuronal damage. In this sense, our therapeutic system presents a superior potential for GBM therapy than CPT analogues already approved for clinical use.

## 2 Materials and Methods

### 2.1 General

All reagents and solvents were purchased from Sigma-Aldrich except for CPT from ABCR and HPLC solvents (HPLC grade from Scharlab or LC/MS grade Optima from Fisher).

Reversed-phase high performance liquid chromatography (RP-HPLC) analysis was performed on an Agilent 1220 Infinity LC coupled to a fluorescence detector 1260 Infinity with an analytical column (Mediterranean Sea C18, 5 μm, 100 mm × 21 mm). The products were eluted utilizing a constant solvent mixture (CH_3_CN/H_2_O–TFA, pH 4.5, 50:50 v/v) at 0.8 mL/min. NMR spectra were recorded on a Bruker AV300 Ultrashield spectrometrer. ^1^H NMR spectra were acquired at 300 MHz employing pulses of 15 μs and a recycle time of 1 s. Data for ^1^H spectra are reported as follows: chemical shift, multiplicity (s = singlet, d = doublet, dd = doublet of doublet, t = triplet, dt = doublet of triplet, m = multiplet), and integration. To obtain ^13^C spectra 9 μs pulses at 75 MHz were applied with a recycle time of 2 s. Both ^1^H and ^13^C experiments were carried out using tetramethylsilane (TMS) as chemical shift reference. Quadrupole time-of-flight (Q-ToF) mass spectra were recorded on an Acquity UPLC Waters coupled with Xevo QToF MS with an Acquity UPLC BEH C18 (1.7 μm, 50 mm × 21 mm) column and using positive electrospray ionization. The products were eluted utilizing a linear gradient solvent mixture CH_3_CN (0.01% HCOOH)/H_2_O (0.01% HCOOH) at 0.3 mL min^–1^ (0–13 min, 80:20; 13–17 min, 0:100; 17–20 min, 80:20). All data collected in centroid mode were acquired using Masslynx software (Waters Corp.).

Cell lines and primary culture cells were incubated at 37 °C under a humidified atmosphere of 5% CO_2_. Rat C6 glioblastoma cells, human U87 and human U251 glioblastoma cells, and rat cortex primary culture cells were used to evaluate CPT-ALA anticancer activity and toxicity at nontumor tissues. All glioblastoma cell lines were purchased from American Type Culture Collection (ATCC, VA, USA). Cell lines were seeded in 96-well culture plates in a final culture medium volume of 200 μLwell, using the following seeding densities; C6 and U87 25 000 cell/mL and U251 50 000 cell/mL. C6 cells also were seeded in 12-well culture plates in a final culture medium volume of 1 mL/well. All cell lines were maintained using DMEM (1×) (GIBCO by Life Technologies) supplemented with 10% fetal bovine serum (FBS-Biowest) and penicillin and streptomycin (Gibco by Life Technologies) 1:100 (v/v).

Rat cortex primary cells were isolated from Sprague Dawley rat embryos at day E17-E18. Mechanically dissociated tissue was added and incubated at 37 °C for 15 min with DMEM and trypsine for chemical dissociation.^31^ Both cellular cultures were originated from cortical cells: isolated astrocytes culture and cortical cells culture were mostly composed of neurons and astrocytes. The use of both cultures allowed us to determine the effect of CPT-ALA in precursor cells to GBM and other CNS cells. Cortical cells obtained with this process were maintained in different culture mediums with the final purpose to obtain both cortical cell culture and astrocytes cell culture. 96-well plate pretreated with poly-d-lysine (PDL, Sigma-Aldrich) and laminine (Sigma-Aldrich) were used to seed both cells types. Cortical cells were seeded with density 65 000 cell/mL and maintained in Neurobasal medium (GIBCO by Life Technologies) supplemented with 2% FBS, 2% B27 growth factor (GIBCO by Life Technologies), 0.4% Glutamax (GIBCO-Invitrogen), and 0.4% penicillin and streptomycin 1:100 (v/v). Astrocytes were seeded with a seeding density of 60 000 cel/mL and maintained in the same cortical cell medium culture, supplemented with 10% FBS and without B27.

All animal experiments were approved by the Ethic Committee of Universidad Miguel Hernández (Elche, Spain) according to the directive 2010/63/EU of the European Parliament and of the Council and the RD 53/2013 Spanish regulation. Sprague Dawley and Wistar rats were obtained from Animal Experimental Service (SEA) of Universidad Miguel Hernández. Animals were maintained at room temperature in a 12 h light and 12 h dark cycle and fed *ad libitum*. Twenty Sprague Dawley rat embryos at day E17-E18 were used to isolate cell cortex primary culture and astrocytes culture. Eight female Wistar rats at 3–4 months old, weighing 200–250 g, were used to create an orthotopic model of GBM.

### 2.2 Synthesis of Camptothecin-20-*O*-(5-aminolevulinate)

First, *N*-*tert*-butyloxycarbonyl-5-aminolevulinic acid (Boc-ALA) was synthesized by optimizing a known recipe.^32^ 12 mL of 5-aminolevulinic acid (1 g, 5.95 mmol) aqueous solution was adjusted to pH 8–10 with aqueous sodium hydroxide (0.1 N). Di-*tert*-butyl dicarbonate (DTBD, 2.76 g, 12.68 mmol) was dissolved in 12 mL of 1,4-dioxane (DOX) and added to the mixture, which was stirred at room temperature for 18 h. The excess of DTBD was removed by washing the mixture with diethyl ether (3 × 100 mL). The aqueous solution was acidified with a hydrochloric acid solution (1 N) to pH = 0.5. Ethyl acetate was added (3 × 100 mL) to extract the Boc-ALA, and the solvent was removed in a rotatory evaporator, obtaining 500 mg (36%).

Boc-ALA (500 mg, 2.163 mmol) was dissolved in 165 mL of anhydrous dichloromethane (DCM) at room temperature, and to this solution were added *N*,*N*′-diisopropylcarbodiimide (DIC) (335 μL, 2.16 mmol), 4-(dimethylamino)pyridine (DMAP) (176 mg, 1.44 mmol), and CPT (251 mg, 0.72 mmol) at 0 °C. Then, the reaction mixture was stirred at room temperature for 16 h under argon atmosphere. The resultant solution was washed with hydrochloric acid 0.1 N, and the solvent was removed in a rotatory evaporator, collecting 792 mg of camptothecin-20-*O*-(*N*-*tert*-butyloxycarbonyl-5-aminolevulinate) (CPT-ALA-Boc, 65%).^33^ Afterward, CPT-ALA-Boc (198 mg, 0.35 mmol) was dissolved in 5 mL of trifluoroacetic acid (TFA/DCM 50:50 v/v) and stirred at room temperature for 1 h. The solvent was removed under reduced pressure and the product was recrystallized from a mixture methanol/diethyl ether (MeOH/DEE 50:50 v/v), obtaining 90 mg of CPT-ALA (45%).

The complete characterization of all synthesized compounds by ^1^H and ^13^C NMR spectra and mass spectroscopy is provided in the Supporting Information. Moreover, stability tests in DMEM and in human serum were carried out in order to check CPT-ALA bioavailability in real conditions. This is described in the Supporting Information.

### 2.3 Protoporphyrin IX Synthesis in Glioblastoma Cells

PpIX synthesis boost by CPT-ALA was tested on the C6 cell line. Cells were seeded in 24-well plates with coverslips at 50 000 cells/well and then incubated in cell medium with 3.6 mM CPT-ALA for 4 h. For comparison, control cells were also incubated in the same conditions with 3.6 mM 5-ALA. Afterward, cells were washed with PBS (1×, pH 7.34), fixed with paraformaldehyde 4% for 20 min at room temperature, and mounted on a slide for further image acquisition using laser confocal scanning microscopy (LCSM) on a Leica TC-SP2-AOBS microscope. PpIX fluorescence intensity was monitored at the maxima for excitation (λ_ex_ = 601 nm) and emission (λ = 405 nm). Moreover, a control was also done with no 5-ALA/CPT-ALA addition

### 2.4 Cell Viability Assay

Cell treatment with CPT-ALA or CPT started 24 h after cell seeding in the case of cancer cell lines and 14 days in the case of cortical cultures. A 1.5 mM stock solution with dimethyl sulfoxide (DMSO) was prepared for every compound, and dilutions were done with cell culture medium (C6, U87, U251, DMEM; cortical cells and astrocytes, Neurobasal). Cells were treated with CPT-ALA or CPT at 0.002–4.8 μg of CPT equiv/mL (CPTeq/mL) concentration range. Cell culture medium was used as cell death negative control (no cell death) and a 30% DMSO solution as a positive control (>99% cell mortality). CPT-ALA and CPT cytotoxicity effect over the cell cycle was studied at two different incubation times, 24 and 72 h. Cells were incubated with drugs at 37 °C under a humidified atmosphere of 5% CO_2_. Cell viability was measured by 3-(4,5-dimethylthiazol-2-yl)-2,5-diphenyltetrazolium bromide (MTT) assay (Sigma-Aldrich). Following cell incubation during 24 or 72 h with CPT or CPT-ALA, the plates were incubated at 37 °C for 3 h with a final concentration of 1 mg of MTT/mL. After incubation, cellular medium with MTT was removed. Then, formazan crystals were dissolved in 100 μL of DMSO and 595 nm absorbance plate was measured in an iMark microplate reader. Absorbance values were normalized with respect to the negative controls and expressed as a percentage by using eq 1:
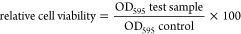
1At least, six independent experiments were performed for every drug concentration, and each experiment was carried out in triplicate. IC_50_ survival data were calculated by nonlinear regression sigmoidal dose–response (variable slope) curve-fiting with Prism 6.0 software (GraphPad).

### 2.5 Apoptosis Rate and Cell Cycle Analysis by Fluorescent-Activated Cell Sorting (FACS)

#### 2.5.1 Apoptosis Rate

Apoptosis caused by CPT-ALA, CPT, or a physical mixture of CPT and 5-ALA (CPT+5-ALA, 1:1 M) was studied on the C6 cell line seeded in 12-well culture plates. Cells were treated 1 day after seeding at DIV 1 with every molecule at 0.002, 0.4, and 1.6 μg of CPTeq/mL for 24 h, at 37 °C under a humidified atmosphere of 5% CO_2_. Then, cells were washed briefly with PBS and trypsinized, subsequently stained with 100 μL of binding buffer containing 5 μL of annexin V-FITC (fluorescein isothiocyanate) and 5 μL of propidium iodide (PI), and incubated in darkness for 15 min at room temperature, according to supplier’s protocol (annexin V apoptosis detection kit, Biotool). Eventually, to it was added 400 μL of binding buffer per sample to neutralize the staining. Samples were kept on ice and analyzed immediately on the FACSCanto system (BD Biosciences). For every concentration 10 000 events were assessed with medium flow. Cells with no staining and treatment were used to delimitate C6 population, whereas cells with no treatment were used as apoptosis and necrosis negative control. Annexin V–/PI– were considered as viable cells. Annexin V+/PI– and annexin V+/PI+ cells were considered as apoptotic cells and annexin V–/PI+ cells as necrotic cells. The obtained data were analyzed with BD FACSDiva software. Four independent experiments were performed for every drug concentration, and each experiment was carried out in triplicate.

#### 2.5.2 Cell Cycle Analysis

To study the action over the cell cycle of CPT-ALA, CPT, or CPT+5-ALA, the cell ratio was determined in each cycle phase by flow cytometry over the C6 cell line. For this sake, cells were seeded in 12 well culture plates and incubated with every molecule at 0.002, 0.4, and 1.6 μg of CPTeq/mL) for 6 or 24 h at 37 °C under a humidified atmosphere of 5% CO_2_. Afterward, C6 cells were fixed with 2% paraformaldehyde and incubated 30 min at 37 °C with 2.5 mg/mL RNase A from bovine pancreas (Sigma-Aldrich) and 2.5 mg/mL PI. Finally, C6 cells were stored in ice and analyzed with FACSCanto system to determine population cell percentage in each cycle phase. For every drug concentration 10 000 events were assessed with low flow. Cells with no staining and treatment were used to delimitate C6 population, whereas cells with no treatment were used as controls to define C6 normal distribution in the cell cycle phases. Data were analyzed through BD FACSDiva software. Four independent experiments were performed for every drug, and each experiment was carried out in triplicate.

### 2.6 *In Vivo* Testing

#### 2.6.1 Tumor Induction

Female Wistar rats of age 3–4 months were pretreated with 1 mg/kg of dexamethasone 24 and 1 h before surgery. Prior to intervention, the analgesia state was induced with a single dose of (40 mg/kg ketamine)/(10 mg/kg xylazine) cocktail using intraperitoneal injection. During surgery, anesthesia was maintained with 3% isoflurane gas mixed with oxygen, and the animal’s body temperature was controlled with an electric blanket. The animal’s head was fixed with a stereotaxic frame. A small craniotomy of approximately 1 mm diameter was performed in stereotaxic coordinates: 1 mm anterior and 3 mm left lateral with respect to the Bregma line.^34,35^ C6 glioblastoma cells were inoculated into the striatum through craniotomy in order to generate an intracranial glioblastoma animal model. 10^5^ C6 cells resuspended in 1.5 μL of PBS were injected with a Hamilton 33-gauge needle to a depth of 4 mm. During the 3 days that followed surgery, analgesic (0.05 mg/kg buprenorphine), anti-inflammatory (0.5 mg/kg meloxicam), and antibiotic (5 mg/kg enrofloxacin) drugs were administrated subcutaneously to improve animal welfare.

#### 2.6.2 *In Vivo* Therapy

Treatment with CPT-ALA started 5 days after tumor inoculation. Animals (*n* = 5) were administered 1 mg of CPT-ALA/kg (corresponding to 0.8 mg of CPTeq/kg) twice a week for 2 weeks, consistent with preliminary work.^36^ For this purpose, a solution of 1 mg of CPT-ALA in DMSO/PBS (1:9 v/v) was prepared and slowly perfused (1 min) through the tail lateral vein with a catheter-gauge-24 at days 6, 9, 13, and 16 after tumor inoculation. Besides, the control group (*n* = 3) was treated with the mixture DMSO/PBS (1:3 v/v) in the same conditions. Animal weight was monitored, and animal welfare was evaluated daily using the Morton and Griffiths scale and facial expressions.^37,38^ According to this test, if animal weight loss exceeds 20% with respect to initial animal weight or if the test score is equal or exceeds 15 points, the animal must be sacrificed to avoid suffering. If the test score is higher than 10 points, euthanasia should be considered. Finally, rats were sacrificed with 40 mg/kg sodic pentobarbital intraperitoneally injected 3 days after the last CPT-ALA administration.

#### 2.6.3 Hystological Analysis

To evaluate CPT-ALA biocompatibility, samples of brain, spleen, liver, kidney, heart, and lung were collected for histological analysis. Tissues were fixed using 4% paraformaldehyde (PFA) during 48 h. Organs were embedded in paraffin and cut with a microtome at 7 μm thickness slices. Sections were deparaffinized and stained with hematoxylin and eosin. Finally, tissue images were analyzed with an Olympus AX70 microscope.

#### 2.6.4 Antitumor Activity

To evaluate CPT-ALA drug antitumor activity, brains were fixed with 4% paraformaldehyde for 48 h and cut using a cryostat at 20 μm thickness slides. Next, an immunohistochemistry assay was done to measure both tumor size and cellular proliferation. Nonspecific staining was blocked for 1 h through 10% bovine serum albumin, and brain slices were permeabilized with 0.5% Triton-X-100. Antibodies anti-Ki67 (Ki67-polyclonal antibody rabbit IgG, Thermo Fisher) and anti-GFAP (glial fibrillary acidic protein antibody mouse IgG, clone GA5, Millipore) were used to target these proteins at nontumoral astrocytes of brain slices. Ki67 and GFAP were detected through secondary antibodies (1:100), respectively, Alexa Fluor 488 conjugated donkey anti-rabbit IgG (Thermo Fisher), and Alexa Fluor 555 conjugated donkey anti-mouse IgG (Thermo Fisher). Cellular nuclei in the slices were stained with HOECHST 33342 (1:300 v/v). Seven random images per slice were taken with a Carl Zeiss Apotome2 microscope to calculate cellular proliferation levels inside the tumor. Total tumor volume was determined by applying eq 2 from the measurement of the two perpendicular radical distances.^39^ Tumor 3D representation was done using Matlab software (MathWorks).
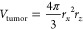
2

#### 2.6.5 TUNEL Assay

TUNEL assay was performed in 20 μm thickness brain slices to compare apoptosis in brain and tumor of the animals with and without CPT-ALA treatment. Brain slices were incubated for 60 min at 37 °C with TUNEL (Roche Molecular Biochemicals). Seven random images per slice were obtained with a Carl Zeiss Apotome2 microscope to determinate cellular apoptosis inside the tumor.

### 2.7 Statistical Analysis

Statistical analysis was performed using arithmetic mean values and error bars of statistical error mean values (SEM). To determine MTT assay significance, *t*-tests and Wilcoxon rank-sum tests were performed. Statistical analyses of all *in vivo* experiments, e.g., TUNEL, proliferation assays, and anticancer activity analysis, were carried out with *t*-tests. Conversely, statistical significance of flow cytometry, e.g., apoptosis and cell cycle analysis, was evaluated using the χ^2^ test. The *t*-tests and Wilcoxon rank-sum tests were performed with Matlab (MathWorks), while the χ^2^ test was carried out using Prism 6.0 software (GraphPad).

## 3 Results

### 3.1 Synthesis of CPT-ALA

**Figure 1:**
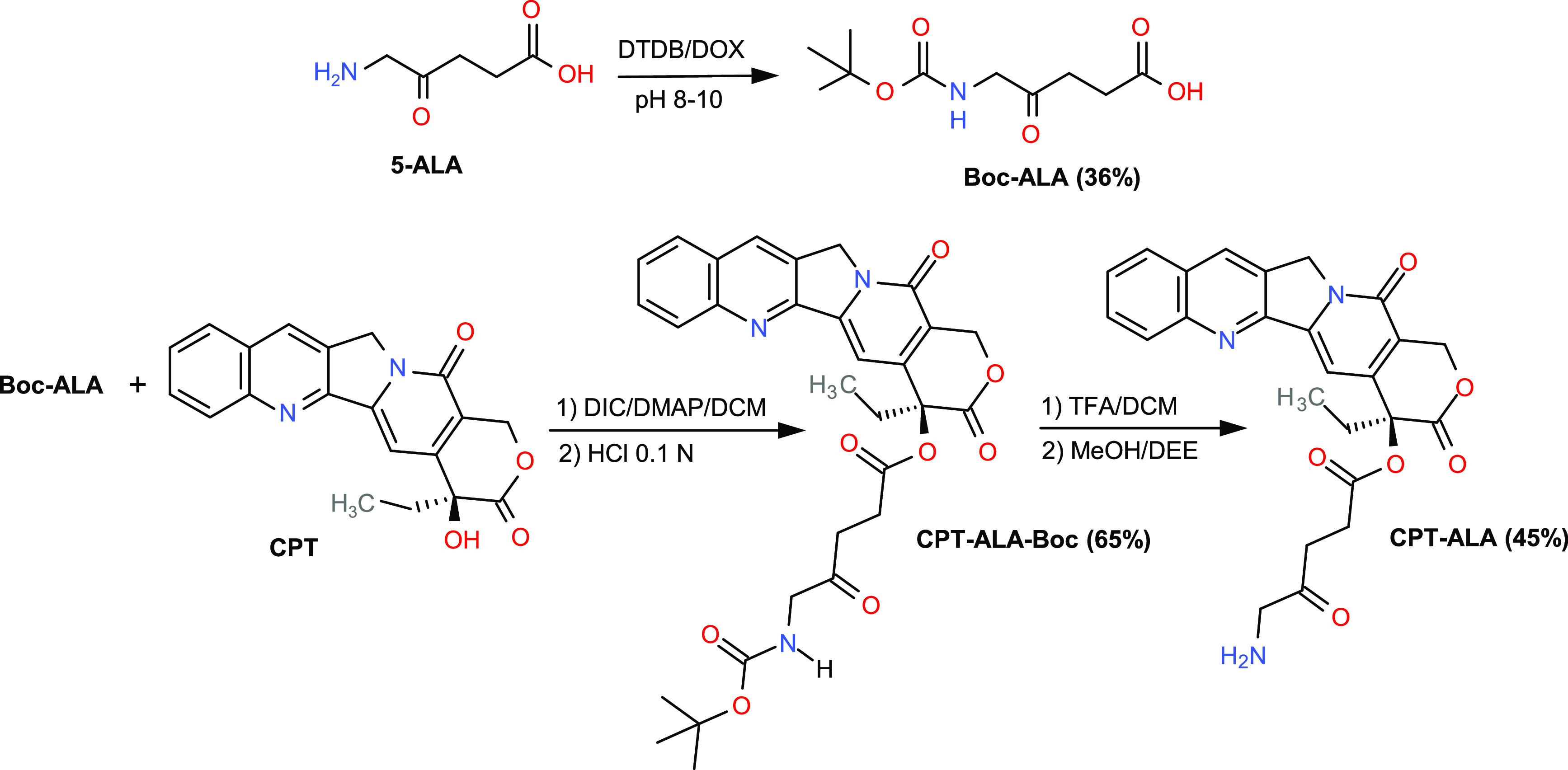
Synthesis scheme for CPT-ALA.

The preparation of CPT-ALA is presented in Figure 1. All compounds were characterized by ^1^H NMR, ^13^C NMR, and mass spectrometry (Q-Tof analysis) (see Supporting Information). The synthesis took place in three steps. First, 5-ALA was reacted with DTBD to give Boc-ALA. This precursor was then conjugated with CPT by esterification of the 20-hydroxyl group. Finally, Boc protection was released with TFA to give CPT-ALA. The complete process runs with 30% global yield, CPT being the only impurity. After a separation step, CPT-ALA was obtained with purity of >99%.

We also carried out stability tests of CPT-ALA in DMEM and in commercial human serum (male AB, Sigma-Aldrich). In cellular medium CPT release was lower than 1% at 24 h (data not shown). Moreover, in human serum no significant CPT release was detected in the first 4 h and, afterward, a slow free CPT concentration increase was observed, which reached about 5% at 24 h (Figure S2). This performance is in line with the stability in physiological fluids of 20-*O*-acylcamptothecin derivatives claimed by different authors^40,41^ and confirms the stability in human serum of these compounds observed previously by our group.^42^

### 3.2 *In Vitro* Study

#### 3.2.1 Protoporphyrin IX Synthesis

CPT-ALA promotes the synthesis of PpIX in glioblastoma cells in a similar way as 5-ALA. This is known on the basis of the PpIX molecular route, which is overexpressed in GBM.^26^ A short incubation time (e.g., 2 h) in C6 cells is therefore enough to achieve successful formation of PpIX *in vitro*, resulting in high red fluorescence intensity (λ = 405 nm), as presented in Figure S3.

#### 3.2.2 Cytotoxicity

**Figure 2:**
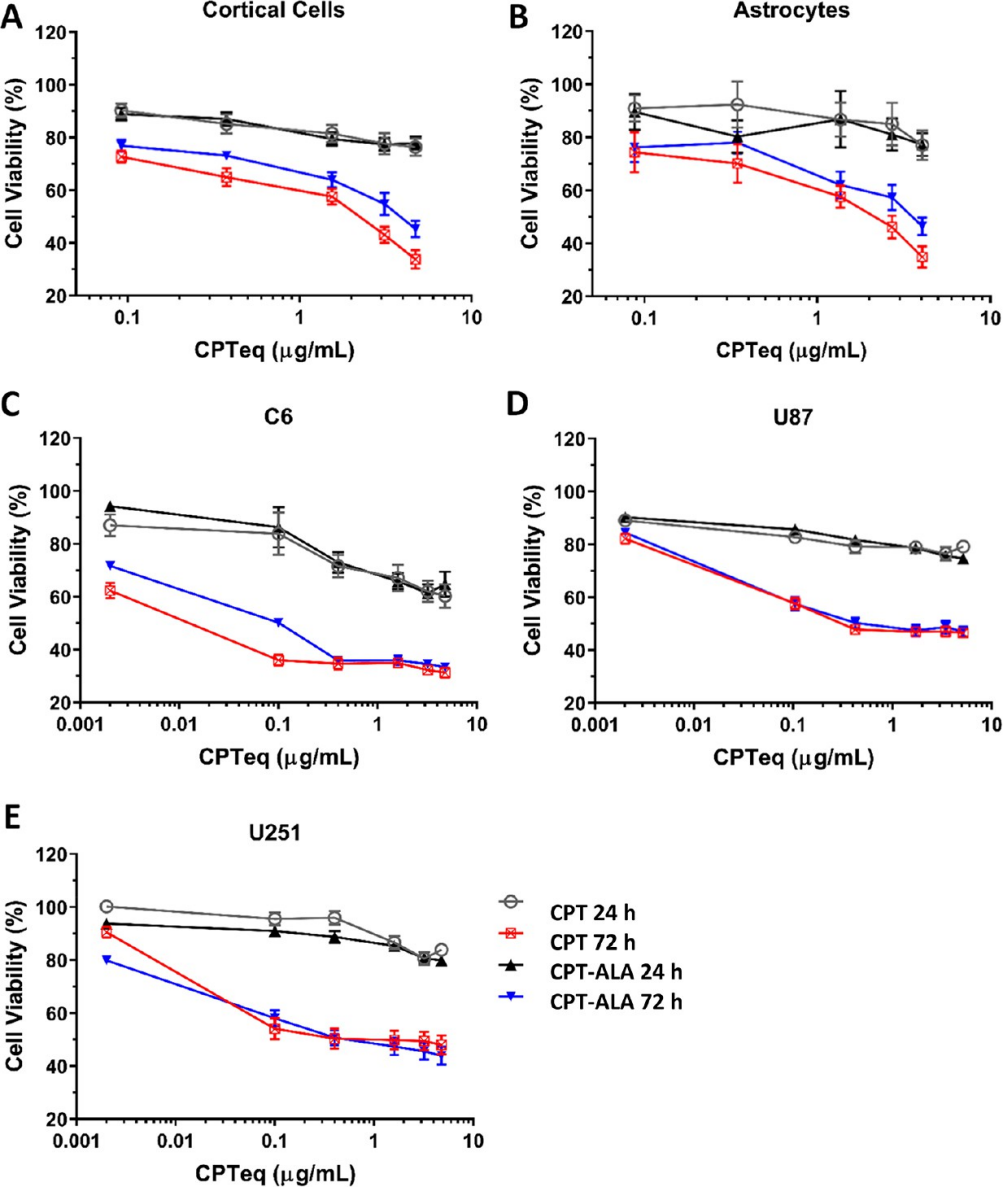
MTT cell viability assays in GBM cell lines and normal CNS cells after 24 and 72 h incubation. Drug concentration is referred to CPT equivalent (lower scale). Cell viability data are expressed as the mean ± SEM: (A) cortical cells (*n* = 10); (B) astrocytes (*n* = 10); (C) C6 cell line (*n* = 6); (D) U87 cell line (*n* = 7); (E) U252 cell line (*n* = 7).

Figure 2 shows quantitative results of cell viability by MTT assay. GBM cell line and primary cortical and astrocytes cultures are represented after the treatment with CPT and CPT-ALA for 24 and 72 h. Very little effect, if any, was observed after 24 h incubation, but a significant increase of cell mortality was observed in all cell types when the treatment was extended up to 72 h (C6, *p* < 0.0001; U251, *p* < 0.0001; U87, *p* < 0.0001; astrocytes, *p* < 0.005; cortical cells, *p* < 0.05), which is consistent with CPT activity over cell cycle. According to some authors 5-ALA has shown some cytotoxicity and genotoxicity over lymphocytes and cancer cells,^43,44^ but we do not think this may be significant in our case, as the cytotoxic activity of CPT is several orders above. No significant differences in the cytotoxic effect over the different GBM cell lines (U87, U251 and C6) were observed for CPT and CPT-ALA. However, cell mortality induced by CPT over normal cell lines is significantly higher than CPT-ALA, the difference becoming especially pronounced in cortical cells (Table 1). This is demonstrated in Figures S4 and S5 (Supporting Information), showing the stronger cytotoxic effect of CPT-ALA with increased concentration when comparing U87 cell line (Figure S4) to cortical cells and astrocytes (Figure S5).

**Table 1:** IC_50_ Values for CPT and CPT-ALA after 72 h Incubation with the Different Cell Lines Tested^a^

cell line	CPT (μg/mL)	CPT-ALA (μg/mL)	*t*
C6	0.016 ± 0.002	0.099 ± 0.015	ns
U251	1.389 ± 1.037	0.914 ± 0.013	ns
U87	2.290 ± 1.740	2.770 ± 1.785	ns
cortical cells	2.733 ± 1.305	7.486 ± 1.400	*p* < 0.01
astrocytes	4.734 ± 1.032	7.521 ± 1.371	*p* < 0.05

a Statistical difference according to Student’s *t* test. ns = not-significant.

#### 3.2.3 Apoptosis Rate

**Figure 3:**
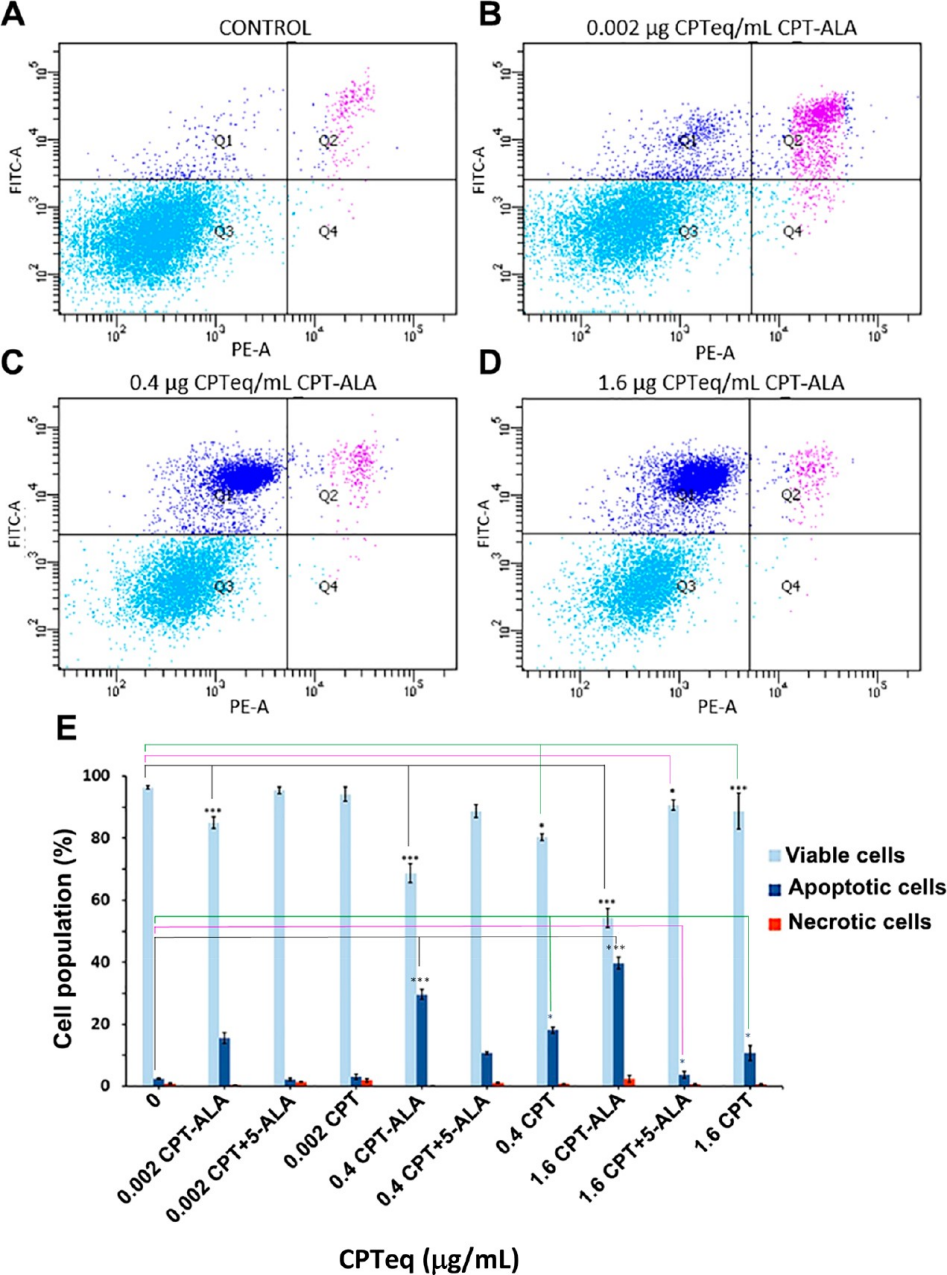
Flow cytometry results after incubation of C6 cells for 24 h with increasing concentrations of CPT-ALA, CPT+5-ALA, and CPT. (A–D) Flow cytometry results after incubation of C6 cells for 24 h with CPT-ALA. *X*-axis: PI fluorescence (PE filter). *Y*-axis: FITC fluorescence. Representative scatter plots (Q1, early apoptotic cells; Q2, late apoptosis cells; Q3, healthy cells; Q4, necrotic cells) of cells with no treatment (A), 0.002 μg of CPTeq/mL (B), 0.4 μg of CPTeq/mL (C), and 1.6 μg of CPTeq/mL (D). (E) Percentage of viable, apoptotic, and necrotic cells treated with CPT-ALA, CPT+5-ALA, and CPT (*n* = 5). Untreated cells were used as negative controls. Data are expressed as the mean ± SD. Viable cells showed significant differences between CPT-ALA (***p < 0.001), CPT+5-ALA (*p < 0.05), and CPT (***p < 0.001) groups and the untreated group. Apoptotic cells showed significant differences between CPT-ALA (***p < 0.001), CPT+5-ALA (*p < 0.05), and CPT (*p < 0.05) groups and the untreated group.

Apoptosis analysis by FC was carried out over C6 cells treated for 24 h with CPT-ALA and stained with annexin V-FITC and PI (PE filter). The cellular population was sorted out into four groups: Q1, early apoptosis cells (annexin V+/PI−); Q2, later apoptosis cells (annexin V+/PI+); Q3, healthy cells (annexin V–/PI−); Q4, necrotic cells (annexin V–/PI+). Figure 3A–D shows how cellular population distribution changed with regard to CPT-ALA concentration, whereas Figure 3e represents the quantitative analysis of these results. A significant increment of apoptosis took place in treated cells (*p* < 0.001) when increasing CPT-ALA concentration (e.g., from 0.002 to 1.6 μg CPTeq/mL) with regard to nontreated cells. In addition, necrosis variation as a function of CPT-ALA concentration was not significant. Consistent with MTT assay results, these data indicate that CPT-ALA drug provokes apoptosis in C6 cells after 24 h incubation. Furthermore, for the sake of comparison, a similar study was carried out with free CPT and the physical mixture of CPT and 5-ALA (CPT+5-ALA, 1:1 M) over C6 cells, and the results are shown at Figure 3E. Here we observed that although all CPT systems significantly increase apoptosis at large dose (e.g., 1.6 μg of CPTeq/mL) after 24 h incubation, CPT-ALA presents a clear superior activity in comparison to CPT and CPT+5-ALA.

#### 3.2.4 Cell Cycle Analysis

**Figure 4:**
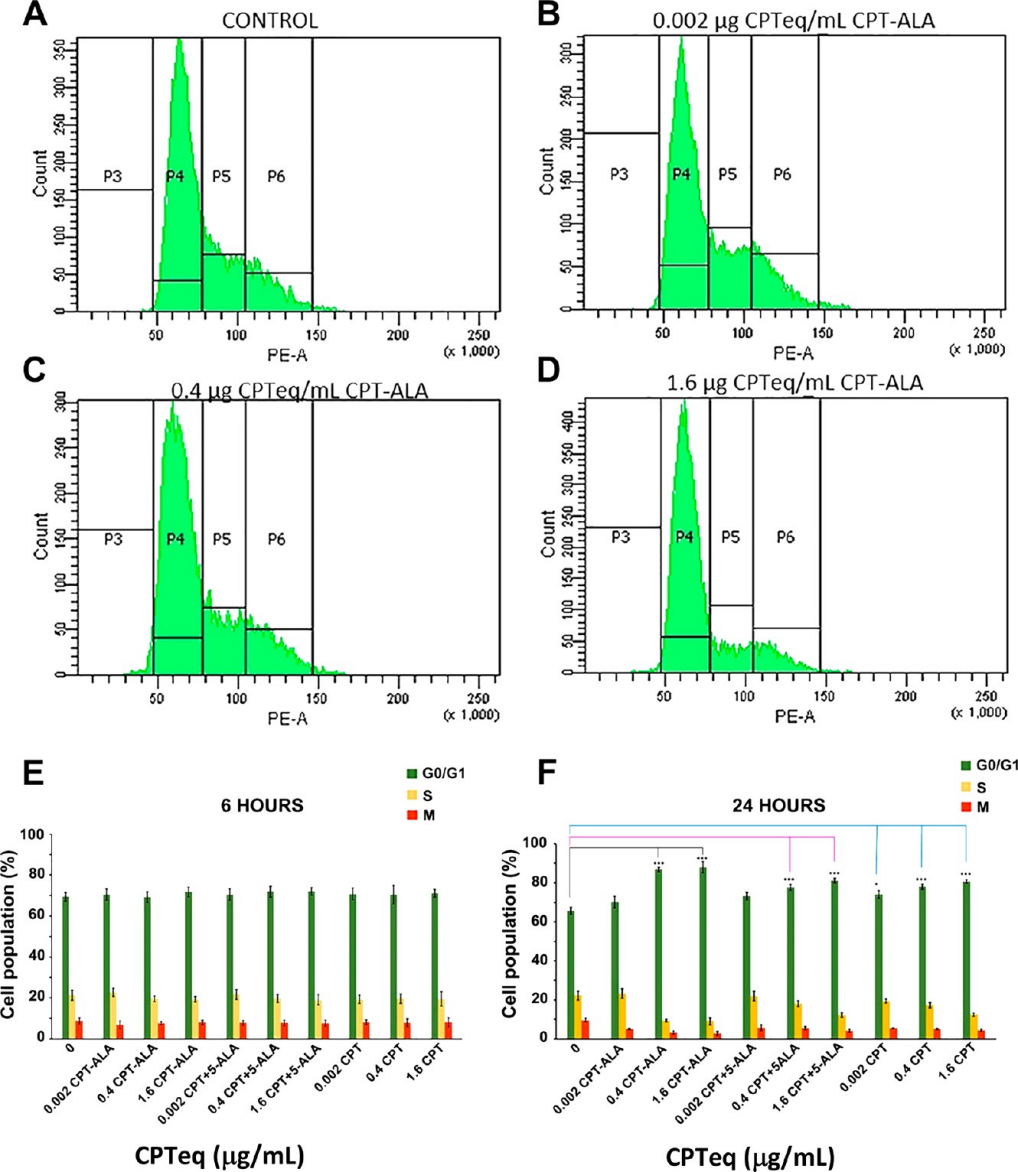
Cell cycle phase study of C6 cells after incubation for 6 and 24 h with increasing concentrations of CPT-ALA, CPT+5-ALA, and CPT. (A–D) Cell cycle phase reports after 24 h incubation with CPT-ALA: control (A), 0.002 μg of CPTeq/mL (B), 0.4 μg of CPTeq/mL (C), and 1.6 μg of CPTeq/mL (D). (E, F) Percentage of cells in each phase of the cell cycle (G0/G1, S, and M) vs CPT-ALA, CPT+5-ALA, and CPT after 6 h incubation (E) and 24 h incubation (F) (*n* = 5). Legend: P4, phase G0/G1; P5, phase S; P6, phase M. Data are expressed as the mean ± SD. Statistical significance (***p < 0.001) compared to the untreated group for CPT-ALA group; statistical significance (***p < 0.001) compared to the untreated group for CPT+5-ALA group; statistical significance (***p < 0.001) compared to the untreated group for CPT group.

The analysis of cell cycle distribution of C6 cells treated with CPT-ALA for 6 and 24 h and stained using PI was carried out by FC. C6 population was sorted out into three subpopulations: G0/G1 phase (P4), S phase (P5), and M phase (P6). Figure 4 shows changes in the distribution of the different cell cycle phases with CPT-ALA concentration. Cell percentage in G0/G1 phase is significantly increased after 24 h incubation with CPT-ALA, whereas cell populations in DNA synthesis (S) and mitosis (M) phases are strongly reduced in the range 0.4–1.6 μg of CPTeq/mL (*p* < 0.001). No significant changes were observed in cell cycle phase distribution after incubation with CPT-ALA for 6 h at the same concentration. In addition, FC assays after PI staining demonstrate that CPT-ALA induced cells to stop in G0/G1 phase and prevented cell cycle progression. Eventually, these results show that the number of cells in a proliferative stage is reduced when the incubation time with CPT prodrug is increased. For comparison, a similar study was carried out with free CPT and with the physical mixture of CPT and 5-ALA (CPT+5-ALA, 1:1 M) on C6 cells, and the results are shown in Figure 4E. Here we observed that all CPT systems significantly increase (*p* < 0.001) cell cycle disruption at phase G0/G1 after 24 h exposure at a wide range of concentration (0.4–1.6 μg of CPTeq/mL), resulting in glioblastoma cell apoptosis.

### 3.3 *In Vivo* Study

#### 3.3.1 Animal Supervision

To ensure animal welfare, weight and pain symptoms were constantly monitored during the experiment. Table 2 shows the evolution of animal weight and Morton and Griffiths scale score in both treated and nontreated groups. Animal weight was kept mostly constant throughout the whole experiment (weight loss was always lower than 20% with regard to the initial value). Face signs and Morton and Griffith’s test results did indicate no intense suffering in any animal. Finally, both groups did not present symptoms such as alopecia, hematuria, or diarrhea.

**Table 2:** Main Parameters Monitored in Animals during CPT-ALA Toxicity Study^a^

parameter	control	CPT-ALA (0.8 mg/kg)
weight loss (%)	0	5
pain	–	+
physical alterations	–	–
behavior disorders	+	+

a According to Morton and Griffiths test score: +++ ≡ 3; ++ ≡ 2; + ≡ 1; – ≡ 0.

#### 3.3.2 Drug Cytotoxicity

**Figure 5:**
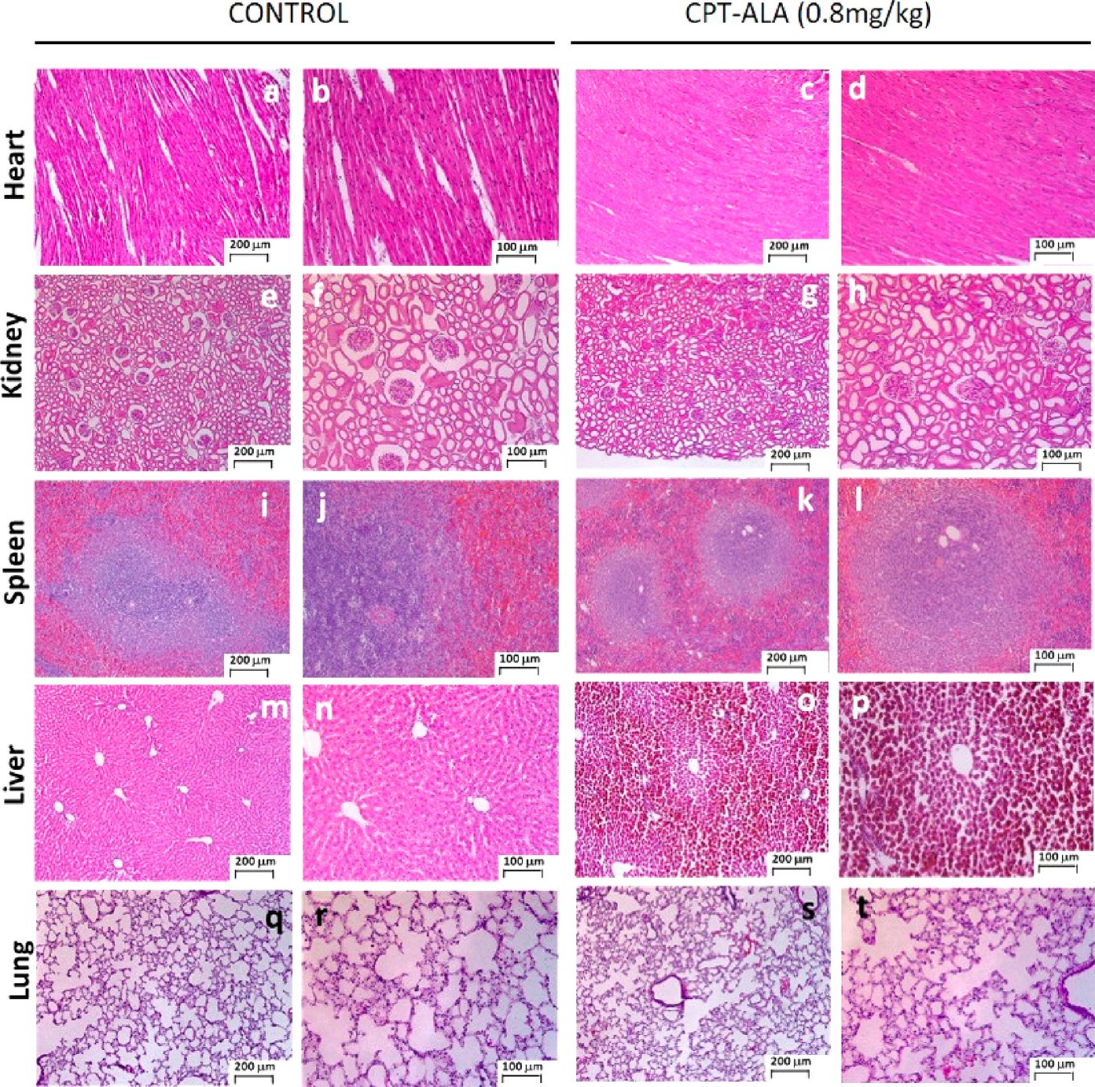
Hematoxylin and eosin staining of histological slices from CPT-ALA and control groups. The therapy was extended for 2 weeks (0.8 mg of CPTeq/kg, 4 doses): (a–d) heart; (e–h) kidney; (i–l) spleen; (m–p) liver; (q–t) lung.

Figure 5 shows representative histological sections of analyzed organs from tumor-bearing rats injected with 0.8 mg of CPTeq/kg for 2 weeks and the corresponding control with no treatment. No structural or cellular abnormalities were observed in the kidney, heart, and spleen of treated animals. Moreover, no alteration or hemorrhages were found in all examined structures of those tissues. Heart histology (Figure 5a–d) did not present changes in myocardium, endocardium, and pericardium between treated and untreated animals. Similarly, kidney histology (Figure 5e–h) did not show any alteration of renal corpuscles and tubules when compared to the control group. Also, spleen histologic images (Figure 5i–l) indicated that the tissue is intact after CPT-ALA administration. No significant immunologic system activity was observed at the spleen, and neither pulp (both red and white) abnormalities nor lymph node was detected.

In addition, histological analysis of the CPT-ALA group did not show any hemorrhage or damage in the liver (Figure 5m–p), but the hepatic tissue showed areas where cells presented pigment deposits inside cells, which were not localized at untreated animals. Furthermore, we have to report that lung alveoli histology showed some hemorrhagic damage in CPT-ALA treated animals (Figure 5q–t), although no chronic issue, as inflammation or fibrosis, was present. Indeed, although these hepatic and lung alteration cases and respiratory and hepatic alterations were not observed, there was no variation in food and water intake, and animals did not present malaise, weight loss, or dehydration symptoms.

#### 3.3.3 Antitumor Activity

**Figure 6:**
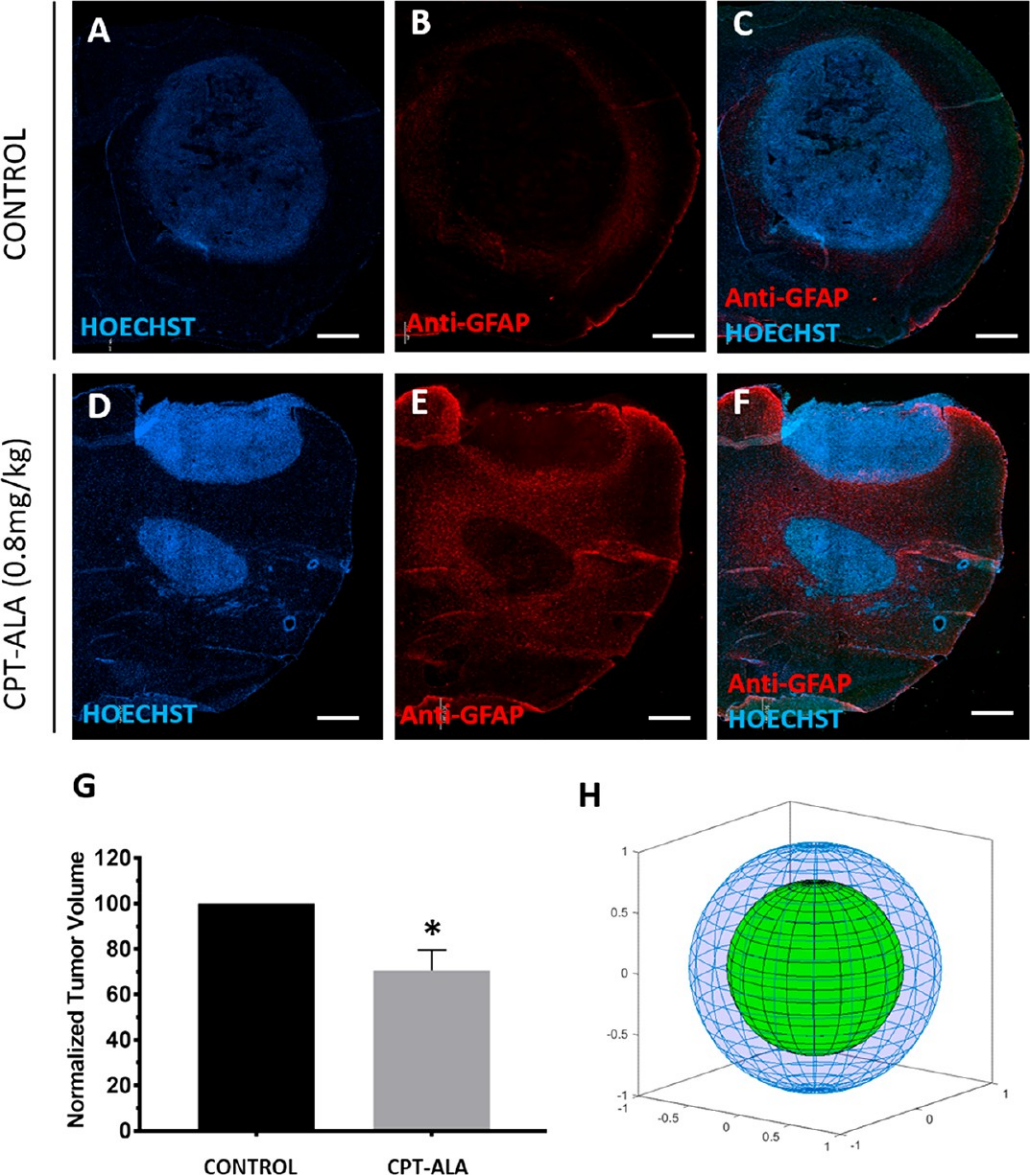
Antitumor activity of CPT-ALA against glioblastoma in Wistar rats. (A–C) Brain slices tissue reconstruction of the control group (*n* = 3). (D–F) Brain slices tissue reconstruction of CPT-ALA group (0.8 mg of CPTeq/kg, 4 doses in 2 weeks) (*n* = 5). Nuclei were stained with HOECHST (blue) and astrocytes with GFAP (red). Scale bar: 1 mm. (G) Normalized tumor volume comparison: control group (*n* = 3); CPT-ALA group (*n* = 5). Data are expressed as the mean ± SEM: *p < 0.05. (H) 3D representation of normalized tumor volume. The blue outer sphere corresponds to the control group, whereas the green inner sphere refers to the CPT-ALA group.

To assess the antitumor effects of prodrug CPT-ALA, we studied its activity against an orthotopic (intracranial) animal model of glioblastoma generated with cell line C6. For this purpose, tumor volume was monitored during 2 weeks in Wistar rats administered with four doses of CPT-ALA (0.8 mg of CPTeq/kg) (CPT-ALA group) or DMSO/PBS (control group). Results (Figure 6) indicated a significant decrease of 30% tumor volume in CPT-ALA injected animals with regard to the control group (*p* < 0.05) (Figure 6G). Modeling of tumor reduction under CPT-ALA administration illustrated tumor recession under CPT-ALA therapy (Figure 6H).

**Figure 7:**
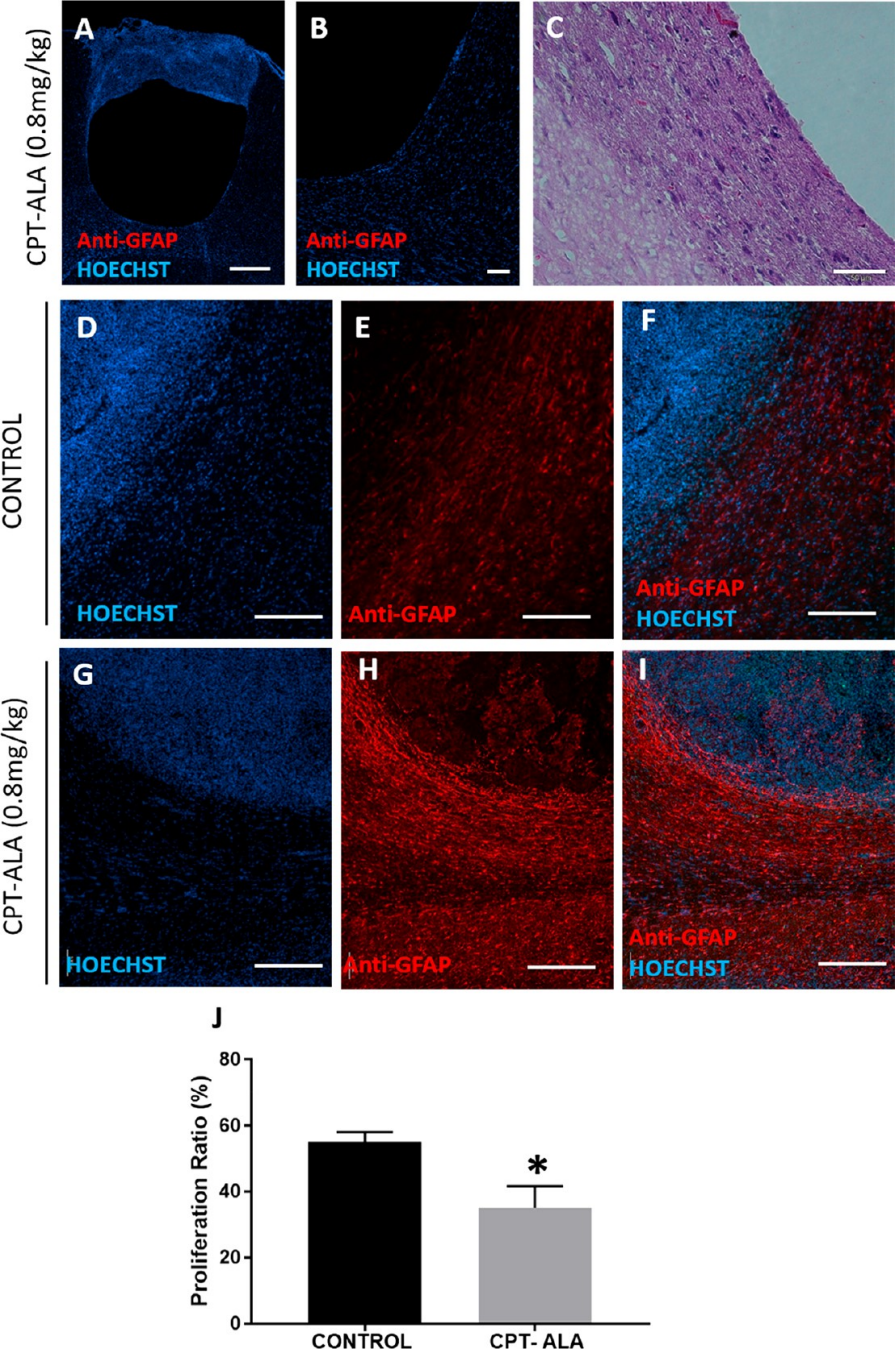
Brain histology and immunohistochemistry after administration of CPT-ALA against glioblastoma in Wistar rats. (A–C) Cystic glioblastoma brain sample reconstruction in a Wistar rat specimen treated with CPT-ALA (0.8 mg of CPTeq/kg, 4 doses in 2 weeks). Scale bar 50 μm. (D–I) GFAP expression in glioblastoma and brain tissue in control group (*n* = 3) (D–F) and CPT-ALA group (*n* = 5) (G–I). Scale bar: 250 μm. (J) Percentage of proliferative cells inside tumor in control group (*n* = 3) and CPT-ALA group (*n* = 5). Data are expressed as the mean ± SEM: *p < 0.05. Nuclei were stained with HOECHST (blue) and astrocytes with GFAP (red) except (C) where nuclei were stained with hematoxylin and cytoplasm with eosin.

Brain histology confirmed no damage over nontumor tissue at the brain or meningeal inflammation in the CPT-ALA group, although we observed the cystic glioblastoma formation in one of the treated animals (Figure 7A–C). In addition, immunofluorescence images (Figure 7D–I) showed that GFAP expression around tumor was higher in the CPT-ALA group than in the control group. This indicated an increase in astrocytes recruitment in tumor surrounding area, with normal surrounding tissue being well-defined in all cases.

Quantification of immunohistochemistry was used to detect Ki67 expression in glioblastoma cells at brain slices, and the results are presented in Figure 7J. Proliferation assay indicated that cells expressing Ki67 decreased 20% in the CPT-ALA group. This demonstrated that the number of cells in division was significantly reduced following CPT-ALA administration with regard to the control group (*p* < 0.05), delaying cell proliferation inside glioblastoma. These data were consistent with the results presented in the cell cycle analysis section 3.2.4, where it was observed that CPT-ALA produced an arrest of glioblastoma C6 cell cycle in the G0/G1 phase.

#### 3.3.4 TUNEL Assay

**Figure 8:**
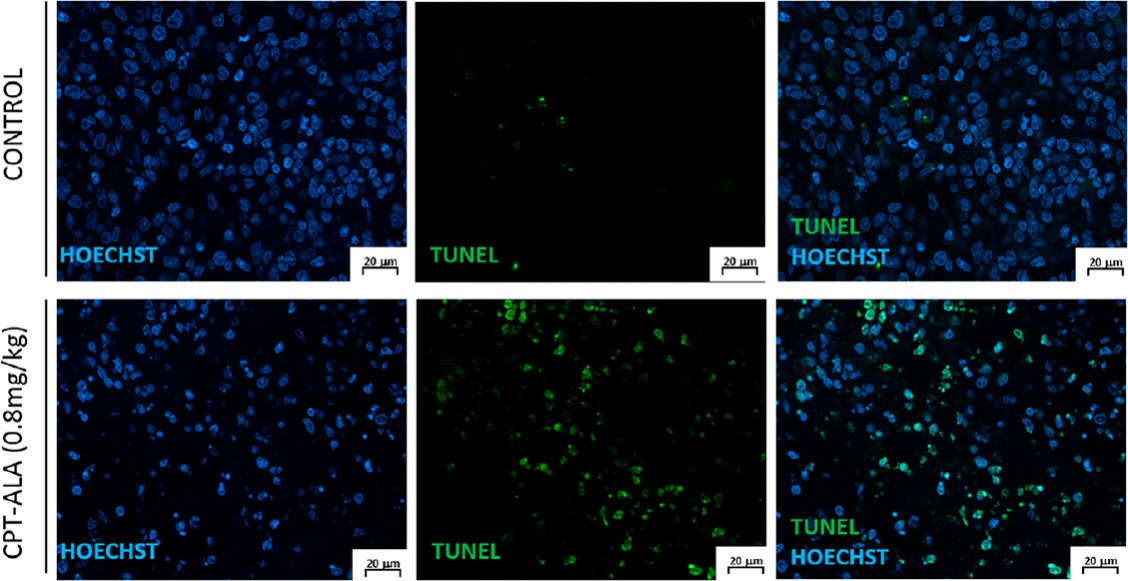
Apoptotic cells stained with TUNEL label (green) in brain slices of control and CPT-ALA treated animals. Nuclei were stained with HOECHST (blue).

Quantification of TUNEL apoptosis assay in brain slices from tumor area is presented in Figure 8. This test showed a significant increase in the percentage in apoptotic cells inside glioblastoma after 2 weeks of treatment with CPT-ALA (control group 15.1%, CPT-ALA group 35.1%, *p* < 0.05). Conversely, no increase of cellular apoptosis was observed in healthy tissue. These data confirm what is presented in the apoptosis rate section 3.2.3, where it was described that CPT-ALA promotes apoptosis in glioblastoma C6 cells after 24 h treatment.

## 4 Discussion

Despite decades of research, GBM remains incurable. Its aggressiveness depends mostly on the protection given by the BBB and its high levels of proliferation. On one hand, BBB blocks the arrival of most drugs to the CNS,^6^ hampering pharmacological therapy and demand for drugs with the potential to go across it. On the other hand, the high rate of division of GBM cells does not only increase its size faster than any other brain tumo,^45^ but also promote a high rate of mutations, which makes GBM quickly resistant to medicines and which contributes to its malignant phenotype.^46^

In this work, a new CPT prodrug, CPT-ALA, is proposed for GBM therapy through intravenous administration. As a powerful inhibitor of DNA topoisomerase I, CPT can stop GBM cell growth.^47^ For this reason, CPT structural derivative irinotecan is a known therapy against GBM, which is usually administrated together with bevacizumab in the recurrent GBM, as well as in patients with unmethylated MGMT gene promoter.^48^ In this context, the conjugation of CPT with 5-ALA should boost the activity of the therapeutic molecule against GBM for two reasons: (i) 5-ALA improves the hydrophilicity of CPT molecule, providing the drug with the ability to go across the BBB, as CPT has shown little efficacy to diffuse through the BBB even when it is formulated with a nanocarrier;^17^ (ii) 5-ALA promotes CPT internalization in GBM cells. Indeed, 5-ALA is a key molecule in the protoporphyrin IX route, which is overexpressed in GBM.^26^ Then, CPT release is expected to take place in the cytosol by the action of specific carboxylases that cleave the ester bond between 5-ALA and CPT.^36^ Consequently, glioblastoma cells present an increasing uptake of 5-ALA, which has been used in photodynamic therapies and as tumor marker during surgical removal.^24^

We studied the anticancer properties of the new prodrug both *in vitro* and *in vivo*. First, viability assays by MTT proved an increase in tumor cell mortality depending on the incubation time for both CPT and CPT-ALA. This is due to the cell needing to be in the cell cycle S phase, thus expressing topoisomerase I, to be vulnerable to CPT activity.^15^ It is noteworthy that no significant differences in the cytotoxic effect were observed for CPT and CPT-ALA over the different GBM cell lines. Indeed, this is not surprising, as *in vitro* models usually provide very favorable conditions for drug intake by cells with accelerated metabolism (e.g., cancer cells).^49^ However, both CPT and its 5-ALA derivative were more toxic in all glioblastoma cell types, but especially in C6 cells, than in cortical cells and astrocytes cultures, and we attribute these results first to the GBM cells accelerated division rate compared to primary cultures. At this point, the doubling time for those cancer cells ranges approximately from 16 h (C6)^50^ to 24 h (U87, U251),^51,52^ whereas astrocytes and cortical cells present doubling times above 4 days.^53^ Furthermore, differences in cytotoxicity between glioblastoma and healthy cells were significantly broadened for CPT-ALA prodrug, due to the promoted cell internalization in cancer cells caused by the 5-ALA moiety. As commented, 5-ALA is a porphyrin metabolized by cells where the heme synthesis pathway is active (e.g., GBM cells but not non-neoplastic CNS cells) to the fluorescent metabolite protoporphyrin IX (PpIX).^24,25^ In this sense, metabolic targeting of 5-ALA takes place over GBM cells,^54,55^ which is enough to promote the superior activity of CPT-ALA over malignant cells with regard to cortical cells and astrocytes cultures. Indeed, 5-ALA targets the prodrug against cells with an increased intake of this molecule (e.g., glioblastoma cell lines) and then reduces the intake by cells with low 5-ALA demand (e.g., normal brain cells).^26^ Hence, the studied prodrug had a strong and similar effect over different GBM cells but limited cytotoxicity over healthy tissue. This has promising clinical applications,^56^ as the lower toxicity should be translated into minimized secondary effects, also allowing the administration of higher CPT-ALA doses.

The results obtained from the *in vitro* viability were further supported by additional studies over the cell cycle. Flow cytometry results at 24 h incubation indicated that CPT-ALA arrested cell cycle of C6 rat glioblastoma cell line in G0/G1 phase by topo-I inhibition. This causes destabilization of the DNA chain, and finally the cascade of cell apoptosis is activated.^57^ Thus, we could confirm that the decrease in cell viability shown in the MTT assays was produced by apoptosis and not by necrosis. However, the comparison with CPT and the physical mixture CPT+5-ALA shows that CPT-ALA promotes clearly better the apoptosis process in the C6 cell line at moderate exposure time (e.g., 24 h). We hypothesize that this is another evidence of the metabolic targeting that the 5-aminolevulinic moiety imposes on the prodrug molecule in glioblastoma cells, which promotes internalization.

*In vivo* testing over Wistar rats has shown that the administration of therapeutic doses of CPT-ALA does not affect the well-being of the studied animals. This is different from what has been observed for other CPT prodrugs, such as topotecan and irinotecan, which normally produce very harmful side effects, like lack of appetite, diarrhea, and alopecia,^13,58^ and is consistent with the rationale of 5-ALA as a molecule that promotes metabolic targeting, increasing CPT selectivity to GBM cells and reducing drug accumulation in other proliferative healthy tissues. Indeed, literature involving *in vivo* testing and clinical trials of topotecan and irinotecan for GBM therapy shows that the standard dose range (∼5–10 mg/kg)^10,59^ is about 1 order higher than CPT-ALA (0.8 mg of CPTeq/kg), which claims the promising potential of this novel prodrug.

Histology results showed no histological damage indicator in kidneys, liver, and spleen after 2 weeks of treatment. However, liver tissue showed pigment deposits inside hepatocytes in the group treated with CPT-ALA, which may consist of heme group accumulation. This would increase the expression of enzymes for the degradation of protoporphyrins and heme group without any harmful effect to the organism.^60^ Moreover, we found some alveolar morphological alterations after CPT-ALA administration, but no breath issue was observed during the treatment. Future studies will be focused on the study of possible long-term respiratory problems, which have also been reported for irinotecan and topotecan.^61^ In this sense, it is noticeable that the treatment with CPT-ALA prodrug has similar side effects as the free 5-ALA molecule, which has already been approved for clinical uses.^60^

Furthermore, the analysis of CPT-ALA anticancer activity in the intracranial GBM rat showed significant differences between control and treated (CPT-ALA) groups, confirming the anticancer activity previously described in *in vitro* assays. Tumor size and cell proliferation values were significantly reduced in the treated animals (30%). Furthermore, an increase of 20% apoptotic cells in tumor tissue was seen, and there was no effect in healthy cortical tissue, which supports the ability of CPT-ALA to induce apoptosis by topo-I inhibition during cell division.^57^

GFAP expression increased around the carcinoma, which indicated the existence of a glial scar encapsulating the tumor.^62^ Indeed, one of the main reasons for recurrence in GBM is its high infiltrative properties in the healthy cortical tissue,^63^ but the appearance of a glial scar following CPT-ALA treatment can reduce the infiltrative capacity of GBM and therefore enhance the success of surgical removing, improving life expectancy by reducing tumor recurrence. In addition, it is also noticeable that in one of the treated animals the tumor evolved into a cystic glioblastoma, as it has been reported that this type of tumor grows more slowly and has a better life expectancy because it has nondiffuse limits.^64^

As an additional remark, it must be taken into account that the BBB increases its permeability in the tumor area due to uncontrolled growth of GBM cells and decreases the expression of ocludin and claudin-1 proteins, which play a key role in BBB structure and organization.^65^ Consequently, it is difficult to confirm experimentally whether CPT-ALA can cross BBB or whether its entry is due to this increase in permeability. At this point, drug diffusion through BBB is usually monitored by HPLC determination in the CNS fluid, but in this case, due to the differences in BBB permeability in the tumor area with regard to healthy tissue, the results obtained might not be representative. Eventually, our *in vitro* and *in vivo* models supported that CPT-ALA can go across the BBB (even considering the possible role of a partially disrupted BBB due to tumor proliferation) and block the reproduction of GBM cells, leading to tumor size recession. At this point, nowadays, GBM treatment remains a challenge due to the difficulty of designing drugs with combined abilities of going through the BBB and killing cancer cells. Although these results are preliminary, we expect to improve tumor collapse by extending the treatment for a longer time and combining CPT chemotherapeutic activity with the photodynamic performance of 5-ALA.

## 5 Conclusion

The success of pharmacological approaches in GBM therapy depends on two crucial steps: (i) the diffusion of the drug through the BBB and (ii) the selective targeting to cancer cells. In this work we have shown by *in vitro* and *in vivo* studies that the novel prodrug CPT-ALA is able to accomplish these points, based on the solubility improvement, the increased permeability of BBB due to tumor proliferation, and the metabolic-based targeting capability of the 5-ALA moiety, which significantly reduces tumor growth with very few side effects. This new prodrug opens the door to combine selective chemo- and photodynamic therapies against GBM in one go due to the specific performance of the 5-ALA molecule.

## Supporting Information Available

Full synthesis and characterization details for CPT-ALA, release kinetics in human serum, protoporphyrin IX fluorescence images, and phase-contrast microscope images of tumor and normal cells (PDF)
