# Supplementary material for: Development of a Prodrug of Camptothecin for Enhanced Treatment of Glioblastoma Multiforme
Source: Mol Pharm. 2021 Mar 1;18(4):1558–72. doi: 10.1021/acs.molpharmaceut.0c00968 (PMC8482753; doi:10.1021/acs.molpharmaceut.0c00968)
Supplement: Supplementary file 1 — mp0c00968_si_001.pdf [file mp0c00968_si_001.pdf]

Supporting Information for

Development of a Prodrug of Camptothecin for Enhanced Treatment of Glioblastoma Multiforme

Elisa Checa-Chavarria,[†] *Eva Rivero-Buceta,*[§] *Miguel Angel Sanchez Martos,*[†] *Gema Martinez Navarrete,*[†] *Cristina Soto-Sánchez,*[†] *Pablo Botella,*[§,]* *Eduardo Fernández*[†,]*

[†] Institute of Bioengineering, Universidad Miguel Hernández, Elche, Spain and Centre for Network Biomedical Research (CIBER-BBN), Avenida de la Universidad s/n 03202 Elche, Spain

[§] Instituto de Tecnología Química, Universitat Politècnica de València-Consejo Superior de Investigaciones Científicas, Avenida de los Naranjos s/n, 46022 Valencia, Spain

1. Synthesis and characterization of camptothecin-20-O-(5-aminolevulinate)

Synthesis of N-tert-butyloxycarbonyl-5-aminolevulinic acid (Boc-ALA). 12 mL of 5-aminolevulinic acid (1 g, 5.95 mmol) aqueous solution was adjusted to pH 8-10 with aqueous sodium hydroxide (0.1 N). Di-tert-butyl dicarbonate (DTBD, 2.76 g, 12.68 mmol) was dissolved in 12 mL of 1,4-dioxane (DOX) and added to the mixture, which was stirred at room temperature for 18 h. The excess of DTBD was removed by washing the mixture with diethyl ether (3 x 100 mL). The aqueous solution was acidified with hydrochloric acid solution (1 N) to pH = 0.5, ethyl acetate was added (3 x 100 mL) to extract the Boc-ALA and the solvent was removed in a rotatory evaporator, obtaining 500 mg (36%).

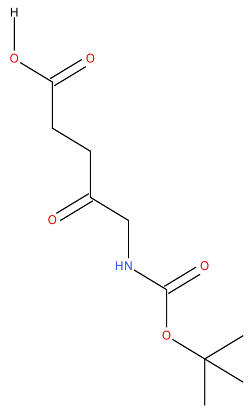

^{1}H-NMR (300 MHz, CDCl$_3$, δ_H): 1.46 (s, 9H), 2.54-2.71 (m, 4H), 3.99 (s, 2H), 5.26 (s$_{br}$, 1H, NH), 10.79 (s$_{br}$, 1H, COOH).

^{13}C-NMR (75 MHz, CDCl$_3$, δ_C): 27.5, 28.2, 34.1, 50.1, 80.1, 156.0, 176.8, 204.8.

Q-TOF MS (ESI, *m/z*) [*M*-H]$^-$ calcd for $C_{10}H_{17}NO_5$, 231.246; found 231.111.

Synthesis of camptothecin-20-O-(5-aminolevulinate) (CPT-ALA). Boc-ALA (500 mg, 2.163 mmol) was dissolved in 165 mL of anhydrous dichloromethane (DCM) at room temperature and to this solution were added *N,N'*-diisopropylcarbodiimide (DIC) (335 µL, 2.16 mmol), 4-(dimethylamino)pyridine (DMAP) (176 mg, 1.44 mmol) and CPT (251 mg, 0.72 mmol) at 0ºC. Then, the reaction mixture was stirred at room temperature for 16 h under argon atmosphere. The resultant solution was washed with hydrochloric acid 0.1 N, and the solvent was removed in a rotatory evaporator, collecting 792 mg of camptothecin-20-O-(N-tert-butyloxycarbonyl-5-aminolevulinate) (CPT-ALA-Boc, 65%).[1]

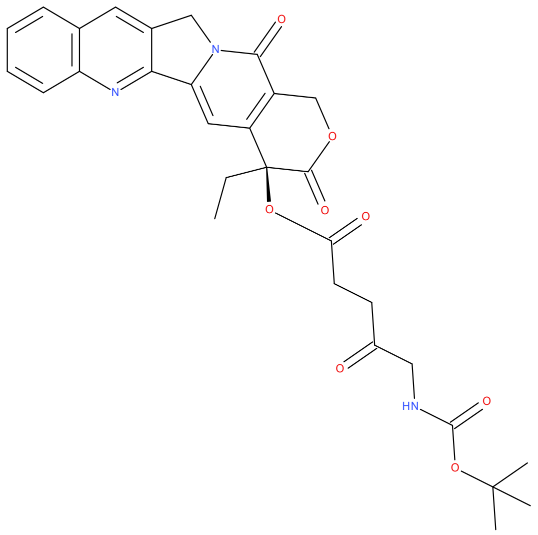

^{1}H-NMR (300 MHz, C_6D_6, δ_H): 0.90 (m, 3H), 1.39 (s, 9H), 2.15 (m, 2H), 2.58-2.81 (m, 4H), 3.77 (m, 2H), 5.30 (s, 2H), 5.48 (s, 2H), 7.06 (t, *J*=6.22 Hz, 1H), 7.12 (s, 1H), 7.72 (m, 1H), 7.87 (m, 1H), 8.18 (m, 2H), 8.70 (s, 1H).

^{13}C-NMR (75 MHz, C_6D_6, δ_C): 7.9, 28.1, 30.4, 33.4, 34.0, 49.4, 49.5, 50.2, 66.3, 78.1, 95.0, 118.9, 127.7, 128.0, 128.5, 128.9, 129.8, 130.3, 131.5, 145.2, 145.9, 147.9, 152.35, 155.7, 156.5, 171.3, 206.2.[2,3]

Q-TOF MS (ESI, *m/z*) $[M\text{-H}]^-$ calcd for $C_{30}H_{31}N_3O_8$, 561.580; found 561.211.

Afterwards, CPT-ALA-Boc (198 mg, 0.35 mmol) was dissolved in 5 mL of trifluoroacetic acid (TFA:DCM 50:50 v/v) and stirred at room temperature for 1 h. Solvent was removed under reduced pressure and the product was recrystallized twice from a mixture methanol:diethyl ether (MeOH:DEE 50:50 v/v), obtaining 90 mg of CPT-ALA (45%). No impurity was detected by HPLC (Figure S1).

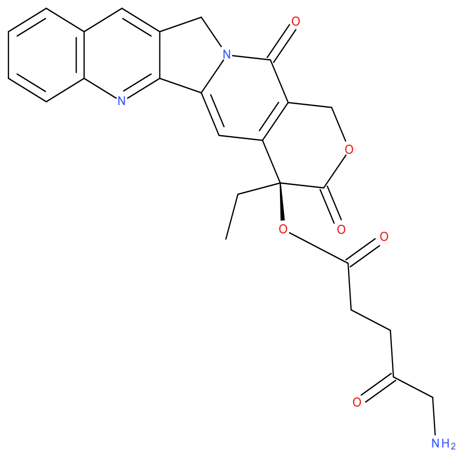

^{1}H-NMR (300 MHz, CDCl$_3$, δ_H): 0.7-0.9 (m, 3H), 1.1-1.3 (m, 2H), 1.3-1.4 (m, 2H), 1.73 (t, *J*=3.53 Hz, 2H), 2.10 (s, 2H), 3.03 (s, 2H), 4.94 (s, 2H), 4.99 (s, 2H), 6.9-7.0 (m, 1H), 7.02 (t, *J*=6.03 Hz, 1H), 7.11 (d, *J*=8.03 Hz, 1H), 7.4-7.5 (m, 1H), 7.6-7.7 (m, 1H,), 8.41 (t, *J*=6.65 Hz, 1H).

^{13}C-NMR (75 MHz, CDCl$_3$, δ_C): 11.0, 27.5, 29.7, 30.4, 30.7, 40.2, 41.6, 66.0, 120.6, 122.5, 125.5, 128.8, 130.9, 132.5, 135.7, 135.8, 136.0, 137.0, 147.6, 149.7, 151.5, 155.2, 157.7, 160.3, 167.8, 202.0.

Q-TOF MS (ESI, *m/z*) [*M*-H]$^-$ calcd for $C_{25}H_{23}N_3O_6$, 477.466; found 477.501.

Area % Report

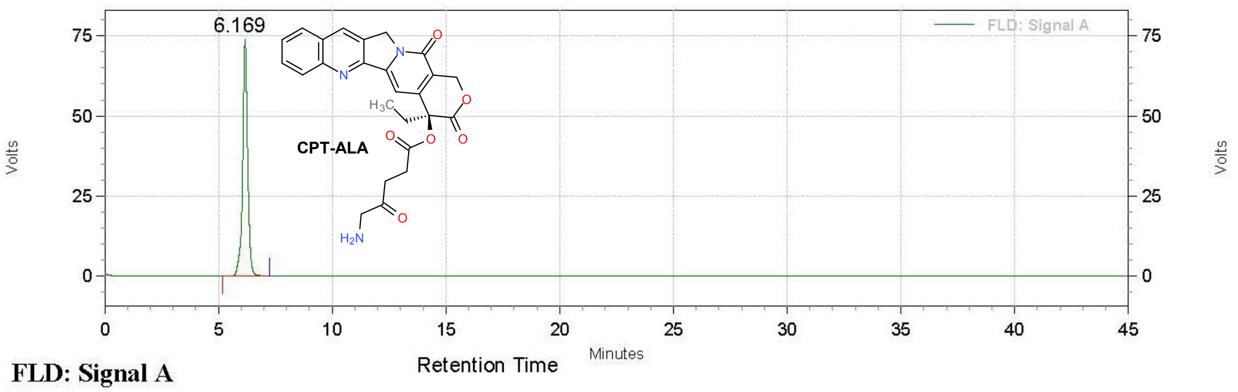

FLD: Signal A
Results

Retention Time	Area	Area %	Height	Height %
6.169	189256202	100.00	12372854	100.00

Figure S1. HPLC report of the as-synthesized CPT-ALA compound.

2. Stability assay

A 1.5 mM CPT-ALA stock solution with dimethyl sulfoxide (DMSO) was prepared. 100 µL of this solution were diluted with 900 mL of DMEM or commercial human serum (male AB, Sigma-Aldrich) and the mixture was incubated at 37 °C in a ThermoMixer®. At the corresponding time, the sample was diluted with 10 mL of methanol at 0 °C and serum proteins were precipitated using a 5% TCA aqueous solution at 0 °C. Then, the supernatant was freeze-dried (-55 ºC, 16 h) and the residue was reconstituted with 1 mL of methanol/HCl (95:5 v/v) solution, and 20 µL of this solution was injected

into the HPLC system. The percentage of free CPT detected in sera samples is presented in Figure S2. Experiments were done by triplicate.

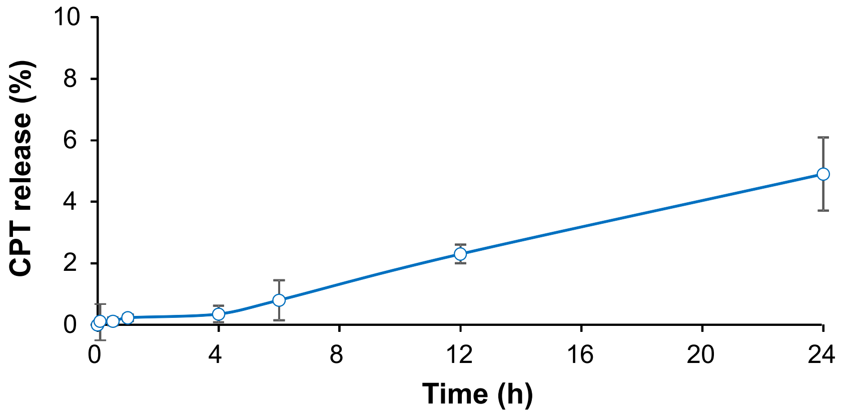

Fig. S2. Release kinetics of free CPT from CPT-ALA hydrolysis in human serum at 37 °C.

3. Protoporphyrin IX synthesis

PpIX synthesis boost by CPT-ALA was tested over C6 cell line. Cells were seeded in 24 wells plates with coverslips at 50000 cells/well, and then incubated in cell medium with 3.6 mM CPT-ALA for 4 h. For comparison, control cells were also incubated in the same conditions with 3.6 mM 5-ALA. Afterwards, cells were washed with PBS (1x, pH 7.34), fixed with paraformaldehyde 4% for 20 min at room temperature, and mounted on a slide for further image acquisition using laser confocal scanning microscopy (LCSM) on a Leica TC-SP2-AOBS microscope. PpIX fluorescence intensity was monitored at the maxima for excitation (λ_{ex} = 601nm) and emission (λ = 405nm). Moreover, a control was also done with no 5-ALA/CPT-ALA addition.

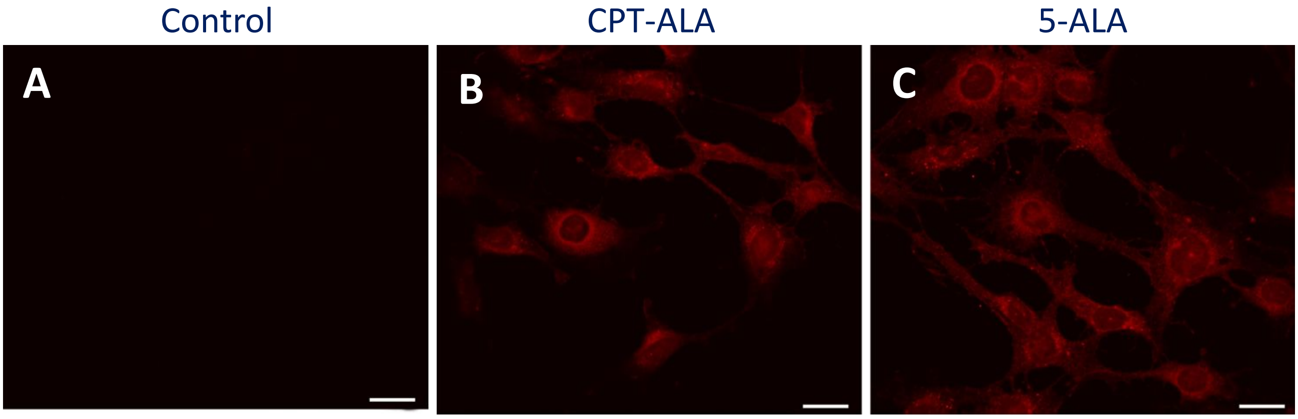

Fig. S3. Protoporphyrin IX analysis after 5-ALA or CPT-ALA incorporation in C6 cells. Fluorescence was evaluated by LSCM 24 hours after application of 3.6 mM CPT-ALA (A) or 3.6 mM 5-ALA (C). Fluorescence emission due to Protoporphyrin IX formation was evaluated at 405 nm (excitation λ_{ex} = 601nm) in C6 cells after 4 h incubation with CPT-ALA or 5-ALA. A negative control represents untreated cells (A). Scale bar: 50 μm.

4. Cell viability assays

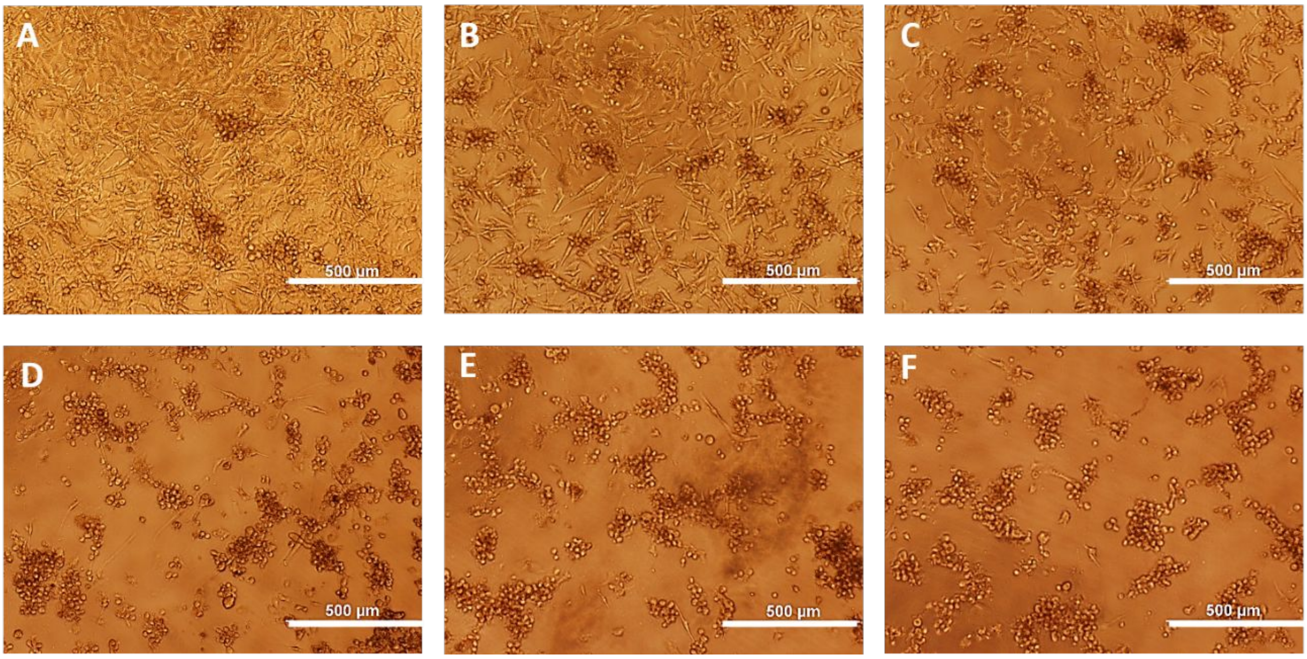

Figure S4. U87 treated-cells images taken by phase-contrast microscope. Cells were treated 72 hours with CPT-5ALA prodrug: A) Control without treatment; B) 0.1 µg CPTeq/mL; C) 0.4 µg CPTeq/mL; D) 1.6 µg CPTeq/mL E) 3.2 µg CPTeq/mL F) 4.8 µg CPTeq/mL.

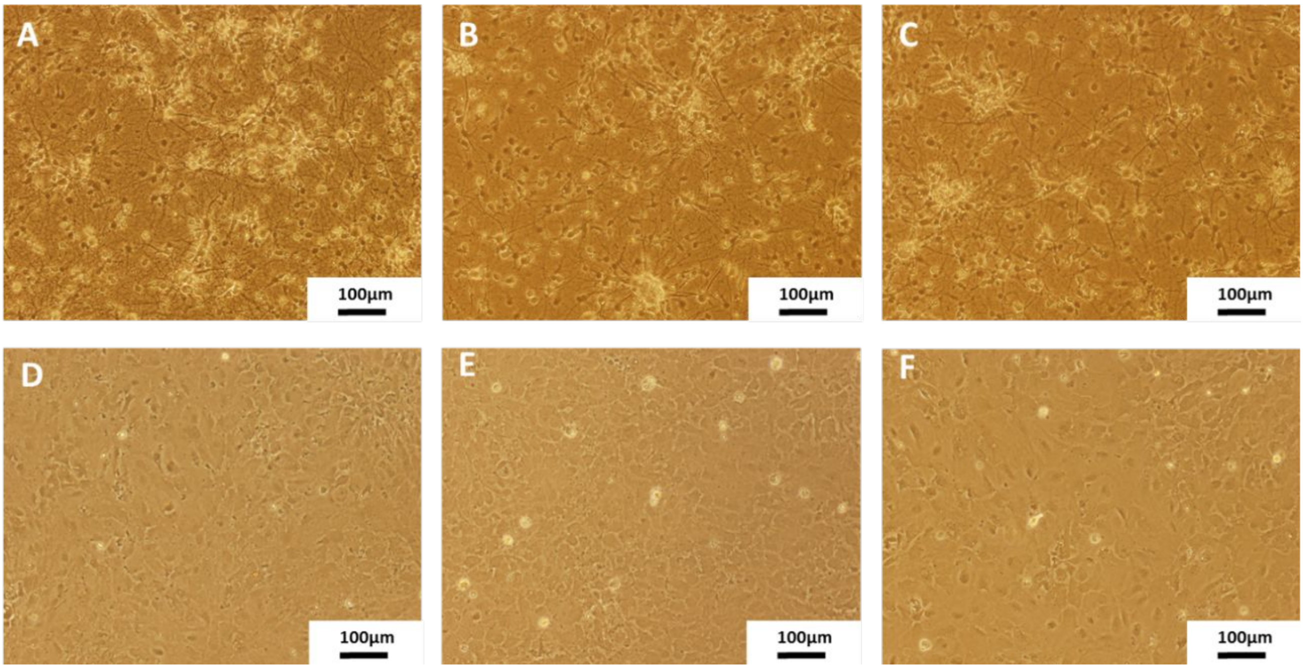

Figure S5. Cortical and astrocytes treated-cells images taken by phase-contrast microscope. Cells were treated 72 hours with CPT-5ALA prodrug. Cortical culture cells: A) Control without treatment; B) 0.4 µg CPTeq/mL; C) 4.8 µg CPTeq/mL. Astrocytes culture: D) Control without treatment; E) 0.4 µg CPTeq/mL; F) 4.8 µg CPTeq/mL.

3. References

(1) Greenwald, R. B.; Pendri, A.; Conover, C. D.; Lee, C.; Choe, Y. H.; Gilbert, C.; Martinez, A.; Xia, J.; Wu, D.; Hsue, M. Camptothecin-20-PEG Ester Transport Forms : The Effect of Spacer Groups on Antitumor Activity. *Bioorganic Med. Chem.* **1998**, *6*, 551–562.

(2) Ezell, Edward L.; Smith, L. L. 1H- and 13C-NMR Spectra of Camptothecin and Derivatives. *J. Nat. Prod.* **1991**, *54*, 1645–1650.

(3) Zhou, H. Bin; Liu, G. S.; Yao, Z. J. Highly Efficient and Mild Cascade Reactions Triggered by Bis(Triphenyl)Oxodiphosphonium Trifluoromethanesulfonate and a Concise Total Synthesis of Camptothecin. *Org. Lett.* **2007**, *9*, 2003–2005.
